# Airway Management of the Right Anterior Segmentectomy through Uniportal video-assisted thoracoscopic surgery (VATS) after left pneumonectomy by an adapted double-lumen endobronchial tube (DLT): a case report

**DOI:** 10.1186/s12871-019-0749-z

**Published:** 2019-05-20

**Authors:** Yang Gu, Ruowang Duan, Xin Lv, Jiong Song

**Affiliations:** The Department of Anesthesiology, Tongji University Affiliated Shanghai Pulmonary Hospital, 507 Zhengmin Rd, Shanghai, China

**Keywords:** Double-lumen endobronchial tube, Pneumonectomy, Ventilation, Isolation

## Abstract

**Background:**

Lung resection after previous contralateral pneumonectomy is rare. We present a case of right anterior segmentectomy despite previous left pneumonectomy, demanding special airway management strategy.

**Case presentation:**

A 48-year-old woman who had left pneumonectomy 2 years ago was scheduled to have the right anterior segmentectomy through uniportal video-assisted thoracoscopy (VATS). A 32-French (Fr) left-sided double-lumen endobronchial tube (DLT) was chosen and adapted. The DLT was intubated into the bronchus intermedius. And the upper lobe can be isolated from the ventilation in the middle and lower lobes when the bronchial cuff’s inflated. The perioperative period was uneventful and the pathological diagnosis was adenocarcinoma.

**Conclusion:**

Lung cancer radical resection was discouraged after previous contralateral pneumonectomy partly due to the challenging ventilation and isolation. With this new DLT adapting and intubation technique showed in this case, the challenging ventilation and isolation that deter the implementation of the operation mentioned above could be solved.

## Background

Contralateral lung resection in postpneumonectomy patient is rare due to its significant perioperative mortality [1]. Thus, the experience in ventilation and isolation for this kind of operation is very limited. Double-lumen endobronchial tube (DLT) has been widely used in thoracic operations to acquire better surgical fields, and the left-sided DLTs are preferred over the right-sided DLTs, because of easier intubation, positioning and effective bilateral suctioning [2]. In postpneumonectomy patients, lung isolation with DLTs could be tricky due to one lung left only. Bronchial blockers (BB) are recommended in selective lobe blockade [3], however, BB is not omnipotent in selective lobe blockade, as in this case, selective right upper lobe blockade entails the balloon to be placed in the right upper lobe bronchus, however, the short right upper lobe bronchus and the angle of the right main bronchus and right upper lobe bronchus would make the placement even more difficult. We have obtained the approval from the Research Ethics Committee and a written patient consent for this report to be published.

## Case presentation

**Fig. 1:**
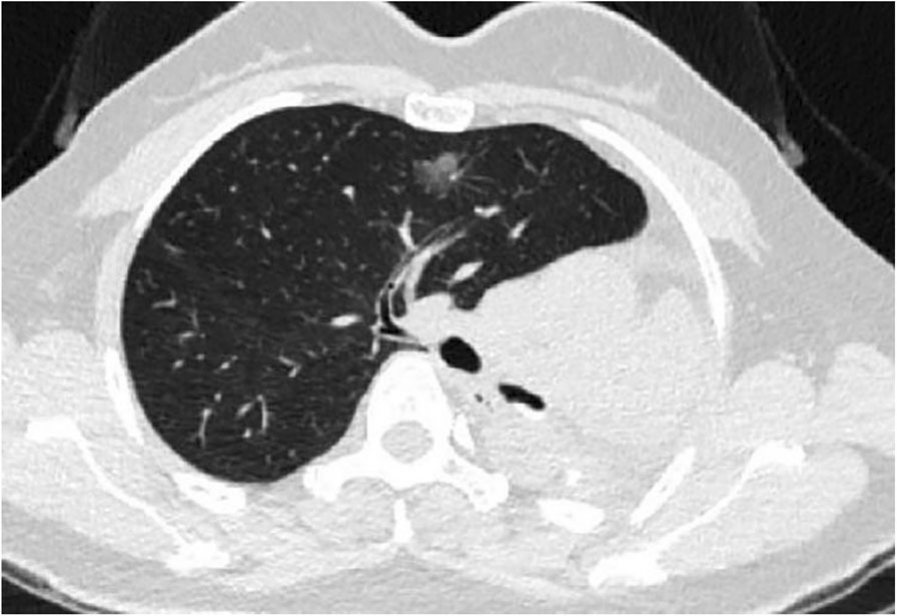
CT (computed tomography) scan of the right anterior GGO (ground glass opacity) and postpneumonectomy

**Fig. 2:**
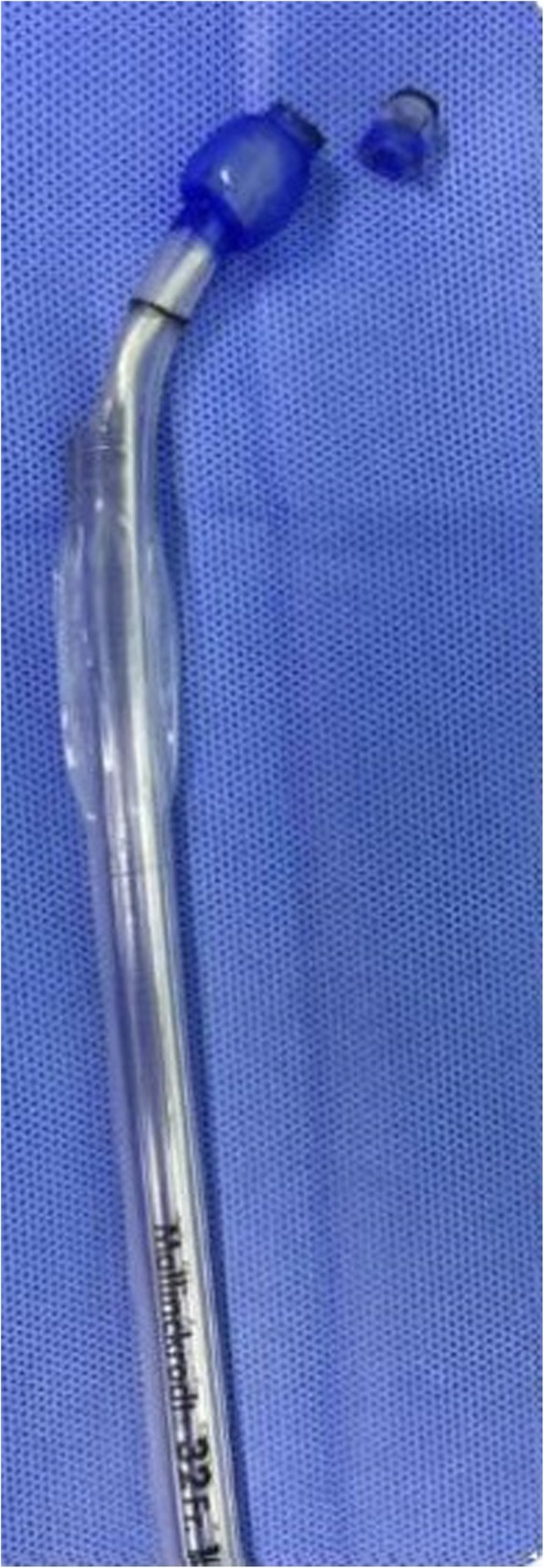
The adapted DLT (double-lumen endobronchial tube)

**Fig. 3:**
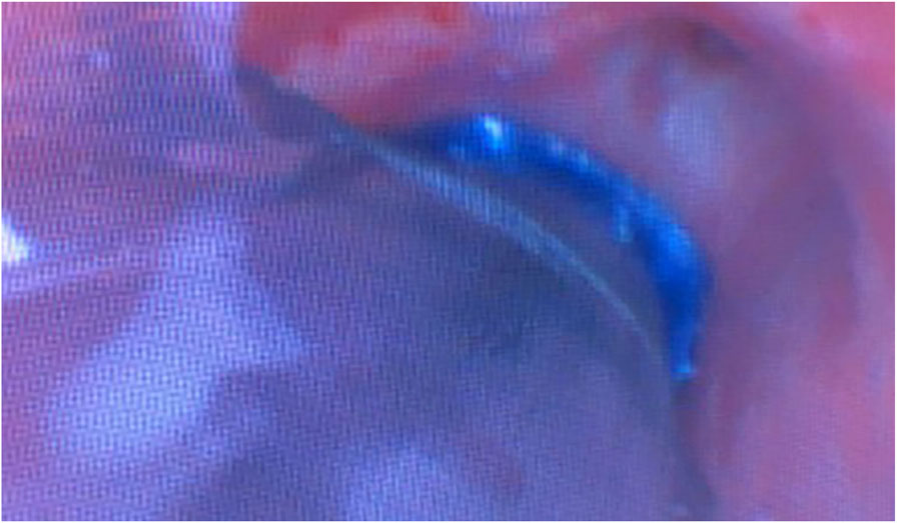
The DLT position achieved by FOB (fiberoptic bronchoscopy)

**Fig. 4:**
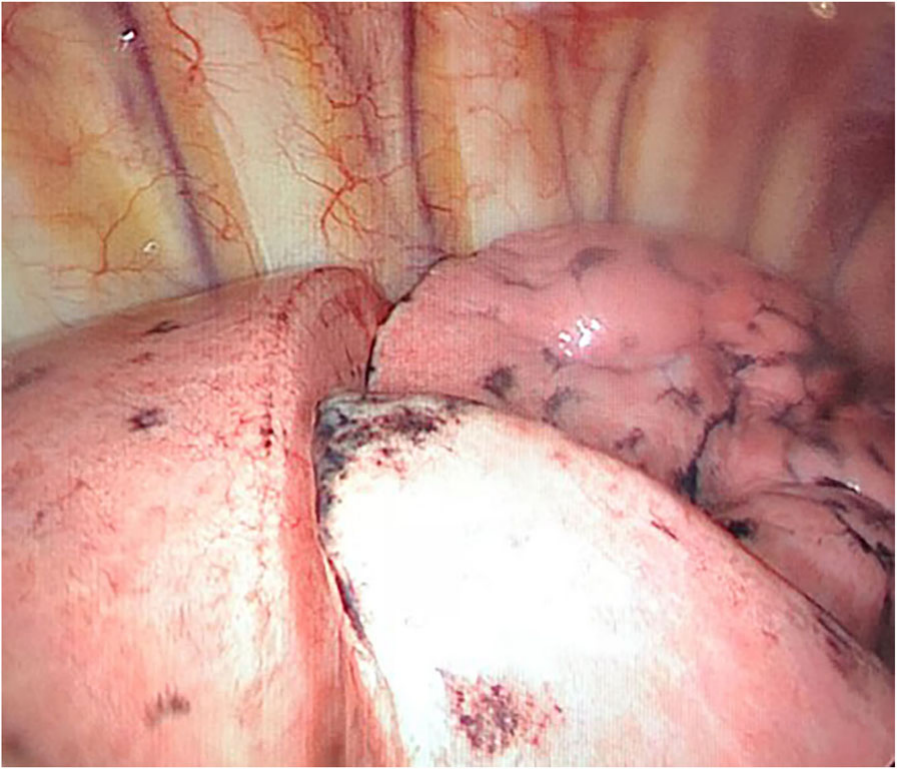
The moment when pneumothorax completed, collapsed upper lobe and ventilated middle and lower lobes

A 48-year old woman (weight 52 kg, height 152 cm, ASA II) was admitted in Sept 18th 2016 because of a ground glass opacity (GGO) which had been detected in the right lung 2 years ago. She had her left pneumonectomy through uniportal VATS owing to the left upper lobe adenocarcinoma invasive to the left main bronchus in Mar 2014. Her pre-operative diagnoses were GGO in the right upper lobe, suspect for malignancy and left postpneumonectomy (Fig. 1). No abnormal findings were detected among other tests, and some of the important figures in the arterial blood gas test were showed as follows: pH 7.44, PaCO_2_ 37 mmHg, PaO_2_ 84 mmHg, SaO_2_ 97.7%. Her pulmonary function test showed FEV1 46.9%, FEV1/FVC 83.3%, and her predicted postoperative FEV_1_% would be close to 44.7%. Although other tests of evaluating cardiopulmonary reserve function and lung parenchymal function were not performed, her regular 3-floor climbing activity was not compromised. The operation was scheduled as right anterior segmentectomy through uniportal VATS under general anesthesia. Routine monitoring was applied and the first data were recorded as follows: body temperature 36.7 °C, blood pressure 123/70 mmHg, heart rate 86/min and Sp0_2_ 98% when the patient was placed in a supine position in the operation room. After the insertion of an 18-gauge intravenous cannula and the right internal jugular vein catheter, intravenous induction was carried out with an injection of midazolam 0.03 mg/kg, sufentanil 0.6 μg/kg, propofol 1 mg/kg, and rocuronium 0.8 mg/kg. Intubation preparation: the patient was scheduled to have the right anterior segmentectomy through VATS after the left pneumonectomy, which entailed us to make a good balance between ventilation and collapse on the right lung only, to make good use of ventilation in the lower and middle lobes, and to produce an effective collapse in the upper lobar. After a prudent study of the following parameters, the diameter of the narrowest part of the tracheal is 11.9 mm, the length of right upper lobe bronchus is 6.2 mm, the angle of the right main bronchus and right upper lobe bronchus is close to 90 degree, the diameter of the bronchus intermedius is 8.8 mm, and the length of bronchus intermedius is about 15 mm, a 32 Fr left-sided DLT was chosen and adapted (Fig. 2). Permission was granted by our hospital ethical committee to adapt the DLTs. The cutting edge of the tube should be smooth and clean, only in this way can we make sure that it won’t do any harm to the airways. One cut (cut just for once) would be best in adapting. Whether the cutting edge is qualified or not can be detected by our sensitive finger tips. And this technique has been proved safe from our experience in tracheal and bronchial operations. The 32Fr left-sided DLTs have been used among short women for left thoracic operations in our facility, and its external diameter is about 10.7 mm, the bronchial internal diameter is about 3.5 mm. But it’s our first time to insert the left-sided DLT to the right for the right lung surgery. As we can see from Fig. 1, the mediastinum has shifted to the left, the intubation should be gentle and carried out by an experienced anesthesiologist in case of any possible injury or even perforation to the former carina. After induction, we performed a FOB (3.0 mm diameter) guided endobronchial intubation with the bronchial cuff into the right bronchus intermedius, and the tracheal cuff’s orifice up against the upper bronchial port (Fig. 3), in this way, the ventilation of the dependent right middle and lower lobes and the collapse of the upper lobar were guaranteed (Fig. 4), thus an appropriate balance between surgical field and oxygenation was achieved, and the blood and sputum from the upper lobe bronchial port can be sucked out. An automatic infusion of propofol and sufentanil combined with manual administration of rocuronium maintained the anesthesia for the operation. And a lung protective mechanical ventilation strategy was taken, positive end-expiratory pressure (PEEP) 5 cmH_2_O, tidal volume (Vt) 4–6 ml/kg, frequency 15–18/min. In the meantime, end tidal CO_2_ and arterial blood gas analysis were recorded to adjust ventilation. The lung recruitment, air leak test and sputum suction went well throughout the operation and the surgery was completed as planned. The patient recovered well after the surgery, so she was extubated in the operation room and sent to the postanesthesia care unit (PACU) for transition, where a routine oxygen supplementation was applied. Oxygenation, ventilation, and circulation were all strictly monitored and no adverse events were recorded in the PACU. She recovered better on the next day follow-up and was discharged from the hospital 6 days later. The pathological diagnosis was invasive adenocarcinoma.

## Discussion and conclusion

It is a case of right lung surgery with previous left pneumonectomy, which entails the resection to be least impairment to the respiratory function and least trauma as well. Uniportal VATS right anterior segmentectomy should be the optimal choice because of its complete removal of the tumor while maximally functional lung kept, and smallest intercostal incision left.

However, this procedure is very challenging in lung ventilation, isolation and maintenance throughout the operation. Except for the way we have successfully implemented, other options could be recommended. First, a single-lumen tracheal tube (SLT) could be intubated into the right bronchus intermedius, this could make use of the ventilation in the middle and lower lobes, yet it also would lead to the upper lobar inflation, unless a detachment of the SLT with the cuff deflated and the ventilator was made before the establishment of pneumothorax. Besides, the blood and sputum from the upper lobe would be a problem since the export would have been blocked by the SLT. Another approach is BB. BB can be served for the purpose of one lung ventilation and selective lobar blockade in VATS, and also its advantages in children and difficult airway placement are widely-accepted [4]. As for this case, however, if BB were chosen, it should be placed into the right upper lobe bronchus, given the knowledge of the anatomical features, it would be more likely placed into some segmental bronchus of the right upper lobe, resulting in inflation in the right upper lobar; besides, even if by any chance, BB were suitably placed, it would have a great probability of being dispositioned given the thought of the short right upper lobe bronchus and the surgical intervention. Although DLT has rarely been taken in selective lobar blockade for lung resections [5], there was a precedent in partial sternotomy [6]. Comparing to partial sternotomy, we believe that lung resection, as in this case segmentectomy is more challenging in ventilation, isolation and drainage. First, we have to make sure the bronchial cuff is intubated into the bronchus intermedius, and it can protect the middle and lower lobes from the upper lobe contamination when inflated, and the bronchial orifice should be above the middle lobe bronchial port to guarantee the ventilation in the middle and lower lobes. But the length of bronchus intermedius varies, and that length was only about 15 mm in this woman while the length of bronchial cuff and the tip included was about 30 mm, so we cut off the tip (about 10 mm without damage to the cuff) and made it clean and smooth without any possible damage to the human body. Still, our approach has some limitations too, the bronchial cuff of this modified DLT was meant to be positioned in the bronchus intermedius, however, the shorter, the easier to be dispositioned; also, skilled adapting and intubation technique, and the recognition of the bronchial anatomy are needed.

But putting the DLT in the expected position doesn’t guarantee a safe oxygenation, considering the middle and lower lobes only for ventilation. Although we assumed it would do, based on her preoperative arterial blood gas results and her daily exercise tolerance, still we asked for ECMO (extracorporeal membrane oxygenation) as an emergent plan in case of extreme low oxygenation. Our lung protective ventilation strategy was a combination of low Vt, high respiratory rate and a small PEEP. It’s been showed in a meta-analysis that in patients without ARDS (acute respiratory distress syndrome), lower Vt is associated with better outcomes, including fewer lung injuries and less pulmonary infection [7]. However, Vt can’t be too low to cause small and distal airways earlier closing and alveoli collapsing, leading to atelectasis, decreased ventilation/perfusion (V/Q) and increased intrapulmonary shunt. It’s been demonstrated that a low Vt combined with an adequate PEEP could promote oxygenation, improve the desaturation status, and decrease the hypoxic lung injury [8].

In conclusion, we have succeeded in managing the ventilation in right middle and lower lobes and the upper lobar isolation by the adapted DLT, enlightening a new technique in selective lobar blockade, and the promotion of radical lung resection after previous pneumonectomy could be benefited.

## Abbreviations

BB: Bronchial blocker
CT: Computed tomography
DLT: Double-lumen endobronchial tube
FOB: Fiberoptic bronchoscope
GGO: ground glass opacity
PACU: Postanesthesia care unit
PEEP: Positive end-expiratory pressure
SLT: Single-lumen tracheal tube
Tidal volume: Vt
V/Q: Ventilation/Perfusion
VATS: Video-assisted thoracoscopic surgery
